# TIGIT is the central player in T-cell suppression associated with CAR T-cell relapse in mantle cell lymphoma

**DOI:** 10.1186/s12943-022-01655-0

**Published:** 2022-09-26

**Authors:** Vivian Changying Jiang, Dapeng Hao, Preetesh Jain, Yijing Li, Qingsong Cai, Yixin Yao, Lei Nie, Yang Liu, Jingling Jin, Wei Wang, Heng-Huan Lee, Yuxuan Che, Enyu Dai, Guangchun Han, Ruiping Wang, Kunal Rai, Andrew Futreal, Christopher Flowers, Linghua Wang, Michael Wang

**Affiliations:** 1 Department of Lymphoma and Myeloma, the University of Texas MD Anderson Cancer Center, Houston, TX 77030 USA; 2 Department of Genomic Medicine, the University of Texas MD Anderson Cancer Center, Houston, TX 77030 USA; 3 The University of Texas MD Anderson Cancer Center UTHealth Graduate School of Biomedical Sciences (GSBS), Houston, TX 77030 USA; 4 Department of Stem Cell Transplantation and Cellular Therapy, the University of Texas MD Anderson Cancer Center, Houston, TX 77030 USA

**Keywords:** Mantle cell lymphoma, CAR T-cell therapy, Relapse, T cell suppression, Myeloid-derived suppressor cells, Immune checkpoint, TIGIT, Cytokines, Chemokines, Soluble receptors

## Abstract

**Background:**

Chimeric antigen receptor (CAR) T-cell therapy using brexucabtagene autoleucel (BA) induces remission in many patients with mantle cell lymphoma (MCL), and BA is the only CAR T-cell therapy approved by the FDA for MCL. However, development of relapses to BA is recognized with poor patient outcomes. Multiple CAR T-cell therapies have been approved for other lymphomas and the resistance mechanisms have been investigated. However, the mechanisms underlying BA relapse in MCL have not been investigated and whether any previously reported resistance mechanisms apply to BA-relapsed patients with MCL is unknown.

**Methods:**

To interrogate BA resistance mechanisms in MCL, we performed single-cell RNA sequencing on 39 longitudinally collected samples from 15 BA-treated patients, and multiplex cytokine profiling on 80 serial samples from 20 patients.

**Results:**

We demonstrate that after BA relapse, the proportion of T cells, especially cytotoxic T cells (CTLs), decreased among non-tumor cells, while the proportion of myeloid cells correspondingly increased. *TIGIT*, *LAG3*, and *CD96* were the predominant checkpoint molecules expressed on exhausted T cells and CTLs; only *TIGIT* was significantly increased after relapse. CTLs expanded during remission, and then contracted during relapse with upregulated *TIGIT* expression. Tumor cells also acquired TIGIT expression after relapse, leading to the enhanced interaction of tumor cell TIGIT with monocyte CD155/PVR. In myeloid cells, post-relapse HLA-II expression was reduced relative to pretreatment and during remission. Myeloid-derived suppressor cells (MDSCs) were enriched after relapse with elevated expression of activation markers, including *CLU* (clusterin) and *VCAN* (versican). Extracellular chemokines (CCL4, CXCL9, CXCL13), soluble checkpoint inhibitors (sPD-L1, sTIM3, s4-1BB), and soluble receptors (sIL-2R, sTNFRII) were decreased during remission but elevated after relapse.

**Conclusions:**

Our data demonstrate that multiple tumor-intrinsic and -extrinsic factors are associated with T-cell suppression and BA relapse. Among these, TIGIT appears to be the central player given its elevated expression after BA relapse in not only CTLs but also MCL cells. The acquisition of TIGIT expression on tumor cells is MCL-specific and has not been reported in other CAR T-treated diseases. Together, our data suggest that co-targeting TIGIT may prevent CAR T relapses and thus promote long-term progression-free survival in MCL patients.

**Supplementary Information:**

The online version contains supplementary material available at 10.1186/s12943-022-01655-0.

## Background

Brexucabtagene autoleucel (BA) targeting CD19 is the only CAR T-cell therapy approved by U.S. FDA to treat patients with mantle cell lymphoma (MCL). BA achieved unprecedented efficacy in highly refractory/relapsed patients [1]. However, development of BA resistance is common and the clinical outcomes after therapy relapse are poor with a median survival of only 4 months [2]. Therefore, it is critical to evaluate the characteristics associated with BA responsiveness and resistance in MCL.

Multiple CAR T-cell therapies have been approved for other lymphomas, such as diffuse large B-cell lymphoma (DLBCL) and follicular lymphoma [3, 4], and resistance mechanisms have been investigated [5–8]. CAR T resistance in lymphoid malignancies can be attributed to both tumor-intrinsic and -extrinsic factors [7, 8]. In the tumor cells, mutations or loss of the target antigen CD19 is a contributing factor for resistance [9, 10]. In the tumor microenvironment (TME), decreased CAR T-cell persistence, enhanced CAR T-cell exhaustion, upregulation of CAR T-cell death receptors, the presence of myeloid-derived suppressor immune cells, a trans-differentiation methylation profile, and overexpression of checkpoint molecules (especially TIM3, LAG3, and PD-1) have all been implicated in resistance [5, 11–14].

The factors underlying CAR T-cell resistance in MCL have not been determined, and whether these factors are shared across lymphoma subtypes, such as DLBCL, is not known. Therefore, in this study, we applied unbiased approaches to investigate the potential resistance factors for BA in MCL using longitudinal primary patient samples. The samples were collected at various treatment time points, pre- and post-BA therapy (i.e., during remission or relapse). MCL is a rare disease, but we were able to assess the largest longitudinal sampling to date. We performed extensive single-cell transcriptomic and high-throughput cytokine multiplex profiling to dissect the intracellular and extracellular factors associated with BA MCL responses and relapses. This is the first such study to explore the intracellular and extracellular events at the cellular and molecular level following BA cell therapy in MCL.

## Methods

### Patients and patient sample collection

Patient samples were collected from peripheral blood, bone marrow, apheresis, or excisional biopsy after obtaining informed consent and approval from the Institutional Review Board at The University of Texas MD Anderson Cancer Center. The patient samples were isolated before cryopreservation. The plasma samples were isolated and stored at − 80 °C.

### scRNA-seq/TCR library preparation and sequencing

The 10x Chromium™ Single-Cell 5′ Reagent Kit v2 (PN-1000190, 10x GENOMICS) and Chromium Single-Cell Human TCR amplification Kit (PN-1000252, 10x GENOMICS) were used to perform single-cell separation, cDNA amplification, and library construction for gene expression and TCR repertoire following the manufacturer’s guidelines. The libraries were sequenced as described previously [15].

### scRNA-seq data processing and analysis

**Raw sequencing data processing, quality control, data filtering, and normalization**: These were performed as described previously [15].

**Dimensionality reduction, unsupervised cell clustering, determination of major cell types and cell states**: These were performed as described previously [15].

**Building single-cell trajectory, pathway enrichment, and characterization of cell-to-cell communication networks**: The single-cell trajectory and pathway enrichment were performed as described previously [15]. Cytotoxic score, naïve score, and exhaustion score were defined as the ssGSEA score of corresponding marker gene sets (Cytotoxic: *CX3CR1, PRF1, GZMA, GZMB, GZMH, GNLY, FGFBP2, KLRG1, FCGR3A, GZMK, LYAR, GZMM, TXNIP, FCRL6, NKG7, KLRD1*; Naïve: *TCF7, CCR7, SELL, LEF1, IL7R, LTB*; Exhaustion: *CTLA4, TIGIT, HAVCR2, LAG3, PDCD1*). The iTALK tool [16] was applied to characterize cell-cell communication signaling networks. The built-in database of iTALK tool [16] was used to functionally annotate identified ligand-receptor pairs, and the visualization tool was used to generate circos plots (RRID:SCR_011798). We defined increased interactions as those where the expression of a ligand-receptor pair was upregulated.

### Cytokine multiplex profiling

We measured the levels of 65 analytes and 20 analytes in two separate kits with overlapping sIL2-R and CD40L for 80 patient plasma samples using the ProcartaPlex Human Immune Monitoring 65-Plex Panel [17] (Invitrogen) and a custom 20-plex panel (also generated by Invitrogen) using Luminex xMAP and the data was analyzed by Luminex xPONENT version 3.1 (Bio-Rad Laboratories, RRID:SCR_008426). The analytes in the 65-plex and the custom 20-plex are listed in Supplementary Table S3.

### ELISA for detection of IFNγ, IL-2, and sIL2R

Patient plasma samples or cell culture supernatants were subject to ELISA for detection of IFNγ (430,104, BioLegend), IL-2 (431,804, BioLegend), and sIL2-R (BMS212–2, Invitrogen) according to manufacturer’s instructions.

### Flow cytometry

We performed flow cytometry to detect TIGIT expression on the cell surface of tumor MCL cells and T cells in the tumor microenvironment of patient samples using the following antibodies: anti-CD3-APC (555,342, BD Bioscience), anti-CD19-PE (555,413, BD Bioscience), and anti-TIGIT-FITC (11–9599-42, eBioscience).

#### Statistical analyses

All statistical analyses of single cells were performed using statistical software R v3.6.0. All other analyses were performed using GraphPad Prism (RRID:SCR_002798). Most data are presented as mean ± SD. Comparison of differences between groups were conducted by two-sided two-sample *t*-test. Results were considered statistically significant for *P* < 0.05 (*); *P* < 0.01 (**); *P* < 0.001 (***); and *P* < 0.0001(****).

## Results

### Patient characteristics and clinical responses to BA therapy

**Fig. 1:**
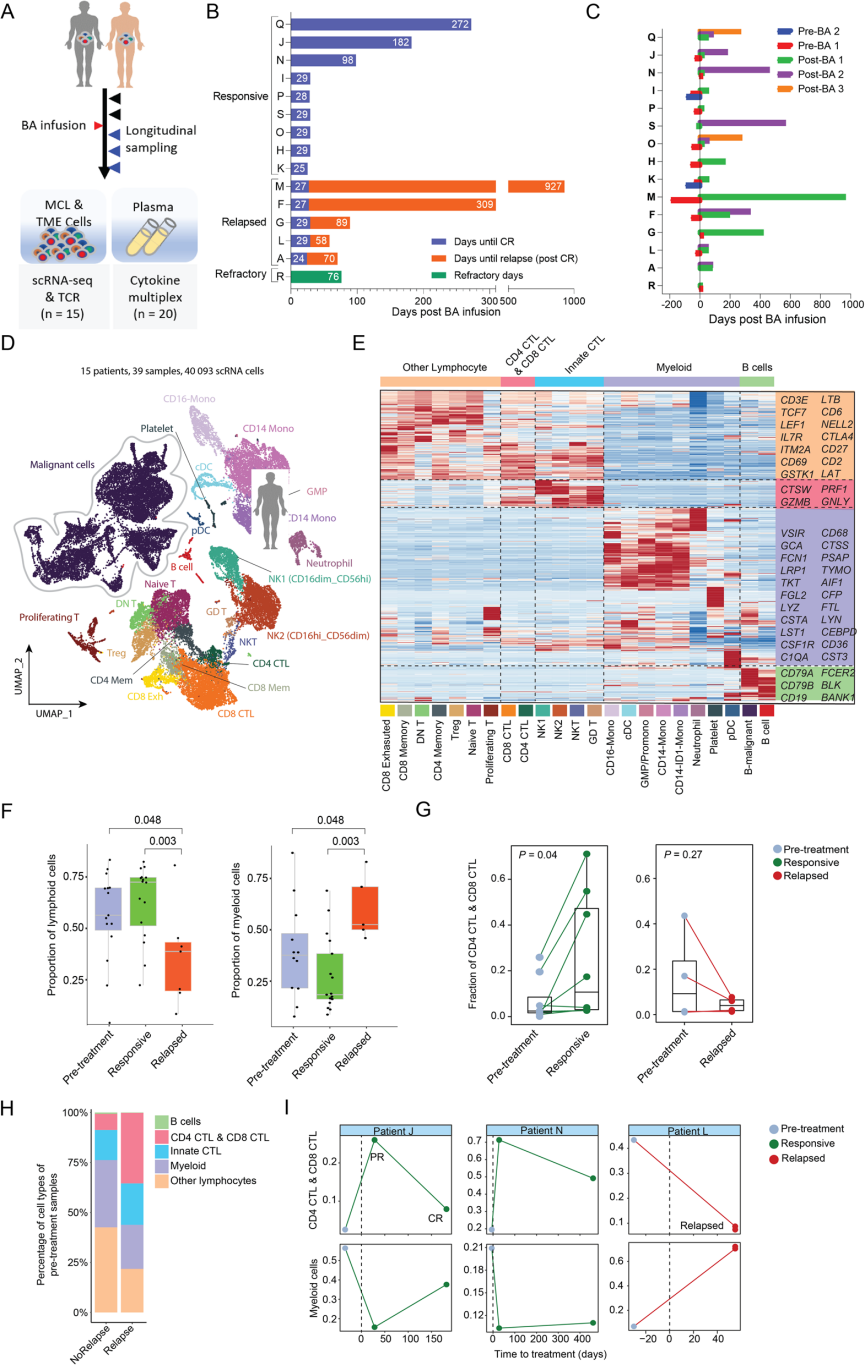
Overall study design and cellular composition of immune cells in the TME. **A** Schematic design for the study. **B** Patient clinical outcome in response to BA therapy. **C** Longitudinal patient sampling for scRNA-seq. **D** UMAP (Uniform Manifold Approximation and Projection) plot of all sequenced cells (*n* = 40,093) that passed QC (Quality Control) for subsequent analyses in this study. Each dot indicates an individual cell; color denotes cell types. **E** Gene expression heatmap analyzed by scRNA-seq. Expression value is the z-score normalized mean expression. **F** Boxplots showing the proportion of lymphoid (left panel) and myeloid (right panel) cells among immune cells. **G** Pairwise comparison of the fraction of CTLs of pre- vs post-treatment samples

We collected longitudinal samples from 15 patients with MCL at various clinical time points before and after BA infusion (Fig. 1A-B). Thirty-nine samples passed quality control (see Methods) and underwent single-cell transcriptome profiling with simultaneous single-cell T-cell receptor (TCR) repertoire analysis (scTCR-seq) (Fig. 1A). Among these, thirty-five samples were collected from peripheral blood (PB), two (L5 and K0) were collected from bone marrow (BM), one (A3) was collected from a lymph node (LN), and one (I2) was collected from the spleen. The patients were grouped into three categories based on their clinical responses after BA treatment: 1) responsive (*n* = 9, patients K, H, O, S, P, I, N, J, and Q), 2) relapsed (*n* = 5, patients A, L, G, F, and M), and 3) refractory (*n* = 1, patient R) (Fig. 1B and Supplementary Table S1). These patients had a median of three prior therapies (range 1–4) and all had failed prior BTK inhibitor (ibrutinib or acalabrutinib) therapy. All patients except patient R had initially attained a complete response (CR) after BA therapy. The responsive group maintained CR with no relapse at the time of last follow up, while the relapsed group achieved initial CR but eventually relapsed (2 months after BA remission for patient A and L, 3 months for patient G, 9 months for patient F, and 30 months for patient M) (Fig. 1B). Additional patient clinical characteristics are summarized in Supplementary Tables S1–2.

### T cell exhaustion and myeloid cell enrichment are associated with relapse after BA

From the 39 longitudinally-collected specimens (Fig. 1C), 40,091 cells with a median of 1859 genes per cell were sequenced by single cell RNA profiling and included in the follow-up in-depth bioinformatics analysis. Among them, 14,719 cells were identified to be MCL cells, and the remaining were non-tumor cells comprising the TME (consisting of 26,272 cells) (Fig. 1D). For TME cells, we identified 10 major lineages, including CD8^+^ cytotoxic T cells (CTLs) (27.3%), CD4^+^ CTLs (12.2%), CD14^+^ monocytes (23.0%), CD16^+^ monocytes (3.8%), natural killer (NK) cells (17.8%), NKT cells (1.1%), and other immune cell populations (14.8%) (Fig. 1D-E, Supplementary Fig. S1A-B). Among these major lineages, four (CD4^+^ T, CD8^+^ T, monocytes, and NK cells) each contained multiple distinct cell states as revealed by sub-clustering analysis based on their subset-specific markers (Fig. 1D-E). In addition, we also detected a total of 12 CAR T cells, which is within the expected range based on the clinical data reported [1]. Because the cell count of these cells was so low, we focused our analyses on the tumor cells and the endogenous TME cell populations.

To identify outcome-associated characteristics, we first checked cellular compositions following BA infusion (Fig. 1E). The fractions of lymphoid cells among total TME cells were significantly decreased after relapse (35%) compared to baseline (55%, *P* = 0.048) and remission (71%, *P* = 0.003) (Fig. 1F left panel and Supplementary Fig. S1C). Conversely, the fractions of myeloid cells were statistically significantly enriched after relapse (Fig. 1F, right panel). Among total lymphoid T cells, the fractions of CTLs (both CD4+ and CD8+) were statistically significantly increased during remission compared to those pre-BA (*P =* 0.04), but decreased after relapse compared to pre-BA (Fig. 1G). These data demonstrate changes in the cellular compositions towards decreased lymphoid cells (especially CD4^+^ CTLs and CD8^+^ CTLs) and increased myeloid cells after relapse.

### The CTLs after relapse are less cytotoxic and overexpress the immune checkpoint molecule *TIGIT*

**Fig. 2:**
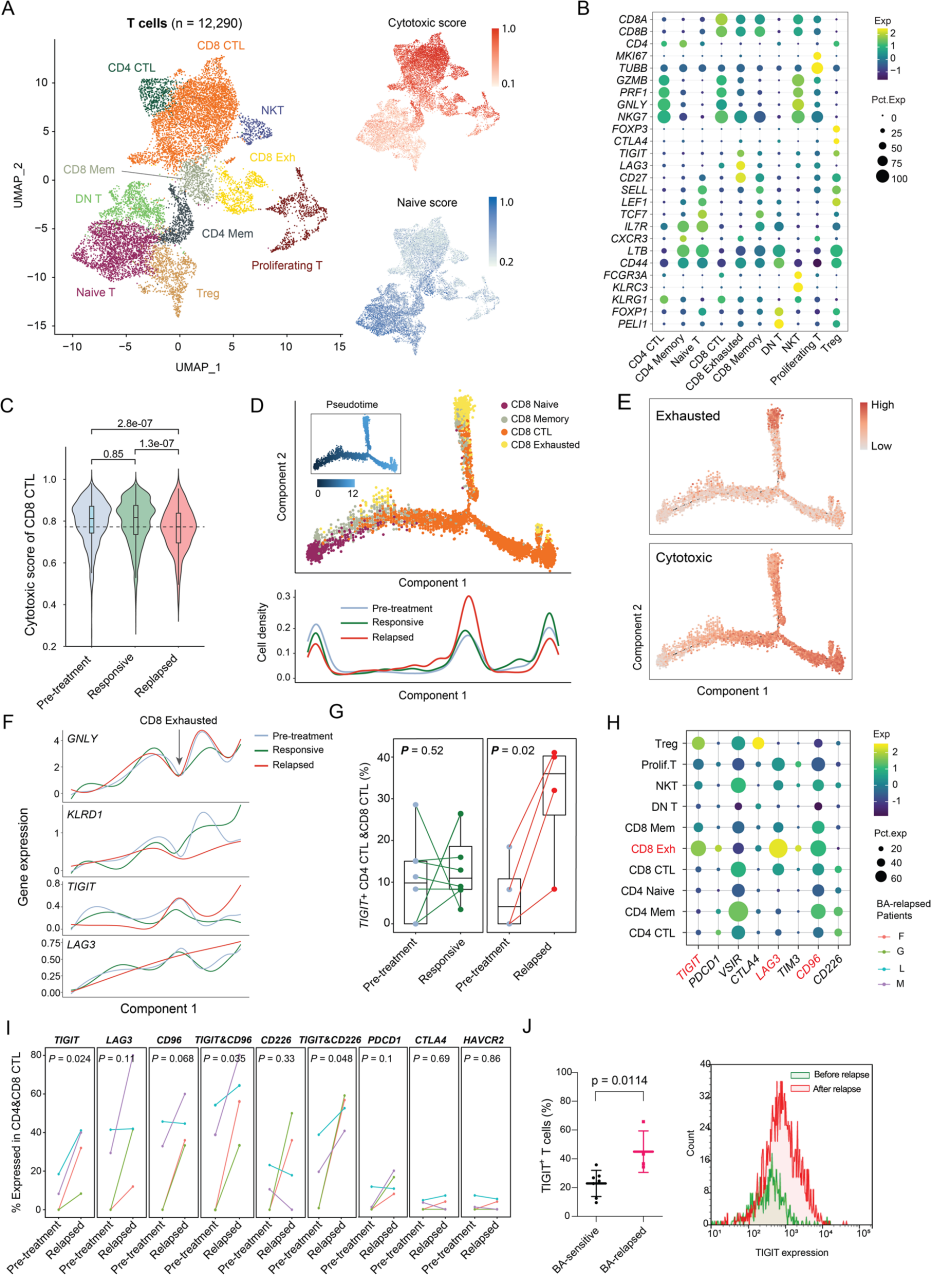
Elevated levels of cytotoxic T cells overexpressing TIGIT post relapse. (**A**) Combined UMAP plots of all T-cell subsets. Each dot indicates an individual cell; color denotes T-cell subsets (left), cytotoxic score, and naïve score (right). (**B**) Bubble heatmap showing marker genes across T cell clusters from **A**. Dot size indicates fraction of expressing cells, colored according to normalized expression levels. (**C**) Boxplots showing the distribution of cytotoxic score of CD8^+^ CTL cells. Mann-Whitney test used to calculate the significances. (**D**) Top, Monocle2 trajectory plot of CD8^+^ T cells. Cell orders are inferred from expression of most differential genes across CD8^+^ T-cell subpopulations. Color is coded by CD8^+^ T-cell subpopulations. Insert visualizes the pseudotime defined by Monocle2. Bottom, cell density relevant to BA response along with component 1 of Monocle2 trajectory. (**F**) Average gene expression of cytotoxic markers and exhaustion markers along with component 1 of Monocle2 trajectory. Loess regression lines of each gene’s expression are shown. (**G**) Pairwise comparison of the fraction of combined CTLs (CD4^+^ and CD8^+^) expressing *TIGIT* among T cells for pre- vs post-treatment samples at the responsive stage (left) or post relapse (right). (**H**) Bubble heatmap showing immune checkpoint molecules across T-cell clusters from (**A**). Dot size indicates fraction of expressing cells, colored according to normalized expression levels. (**I**) Pairwise comparison of the fraction of combined CTLs (CD4^+^ & CD8^+^) expressing immune checkpoint molecules for pre- vs post-treatment samples post relapse. (**J**) TIGIT expression is upregulated on the cell surface of T cells in the tumor microenvironment of BA-relapsed patients (*n* = 4) compared to BA-sensitive patients (*n* = 7) (left panel). TIGIT expression on T cells was assessed after relapse compared to before relapse in a representative patient (right panel)

To understand how T cells are associated with BA relapse, we first investigated the T cell subset compositions. The T cells could be sub-clustered into 10 subsets: naïve T cells, CD4^+^ CTLs, CD4^+^ memory T cells, CD4^+^ Tregs, CD8^+^ CTLs, CD8^+^ memory T cells, CD8^+^ exhausted T cells, DNT cells (CD4/CD8 double-negative T cells), NKT cells, and proliferating T cells (Fig. 2A-B). Based on the cytotoxic/exhaustion/naïve scoring algorithm, the cytotoxicity score was significantly lower in the CD8^+^ CTLs after relapse than it was before BA (*P* = 2.8e-7) and during remission (*P* = 1.3e-7) (Fig. 2C). Trajectory analysis revealed an increased density of exhausted CD8^+^ T cells after relapse (Fig. 2D), while CD8^+^ CTLs were highest during remission and lowest after relapse (Fig. 2D). This correlated well with exhaustion and decreased cytotoxic score (Fig. 2E). The cytotoxic score correlated well with expression of the cytotoxic marker *GNLY*, and the activation marker *KLRD1,* while the exhaustion score was associated with the exhaustion marker *TIGIT*, but not *LAG3* (Fig. 2F). Consistently, expression of *TIGIT* was statistically significantly increased in CD4^+^ CTLs and CD8^+^ CTLs after relapse (4/4, *P* = 0.024) (Fig. 2G, right panel), but not during remission or pre-BA (Fig. 2G, left panel). Of note, the CTLs after relapse also expressed the inhibitory receptors *LAG3* (3/4, *P* = 0.11) and *CD96* (3/4, *P* = 0.068), and only a small subset of the CTLs expressed *PDCD1*, *CTLA4*, or *TIM3* (Fig. 2H-I). This would indicate that higher percentages of.

CD4^+^ and CD8^+^ CTLs acquired expression of *TIGIT* after relapse. Indeed, elevated TIGIT expression was confirmed on cell surface of T cells from BA-relapsed patients compared to BA-sensitive patients (*P* = 0.0144). This suggests that these CTLs are less cytotoxic following BA relapse.

### Endogenous T cell clones expand during remission, but lessen after relapse

To understand how endogenous T-cell clones respond to BA therapy, we tracked T-cell clonal expansion and clearance by scTCR-seq analysis (Supplementary Fig. S2A). The most abundant TCR clones (> 20 cells/clone) were predominantly associated with CD4^+^ and CD8^+^ CTLs (Supplementary Fig. S2B) and the T-cell clone sizes were increased during remission, but decreased after relapse (Supplementary Fig. S2C), suggesting an enrichment of large TCR clones during remission. Indeed, trajectory analysis for the TCR clones revealed that larger clones of CD8^+^ CTLs were positively correlated with the pseudotime progression (Supplementary Fig. S2D), which was consistent with a higher cytotoxic score (Fig. 2D-E). Of interest, a subset of CD8^+^ CTL clones with relatively smaller cell number clustered together with the exhausted CD8^+^ T cells (Supplementary Fig. S2D), suggesting that this smaller subset of CD8^+^ CTL clones were less cytotoxic and resembled the exhausted T-cell clones with respect to their transcriptomes.

### T cells after relapse are functionally deficient

To validate the T-cell functions during BA remission and after relapse, samples from patient F were used, because only this patient had samples available at all treatment stages (before BA, during remission, and after relapse). As a TCR-independent stimulator, phorbol 12-myristate 13-acetate/ionomycin (P/I) induced robust production of IFNγ and IL-2 in T cells expanded from healthy donors, which served as a positive control (Supplementary Fig. S3A). P/I also statistically significantly (*P* < 0.001) induced IFNγ and IL-2 production in the sample during remission, but not in those pre-BA or after relapse (Supplementary Fig. S3B). This indicated that the T cells had the potential for activation during remission, while those pre-BA or after relapse did not. As expected, the TCR-dependent stimulator anti-CD3/CD28 induced robust T-cell expansion of the healthy peripheral blood mononuclear cells (PBMCs) in the presence of IL-2 (Supplementary Fig. S3C-D). However, the T cells collected after relapse failed to proliferate and expand under similar conditions. Furthermore, the relapsed sample failed to induce the robust production of IFNγ, IL-2, and sIL-2R in ex vivo culture as seen in the healthy PBMCs (Supplementary Fig. S3E). These data further support that the T cells collected after relapse were likely functionally deficient.

### Monocytes and neutrophils increase after relapse and display reduced human leukocyte antigens class II molecules

**Fig. 3:**
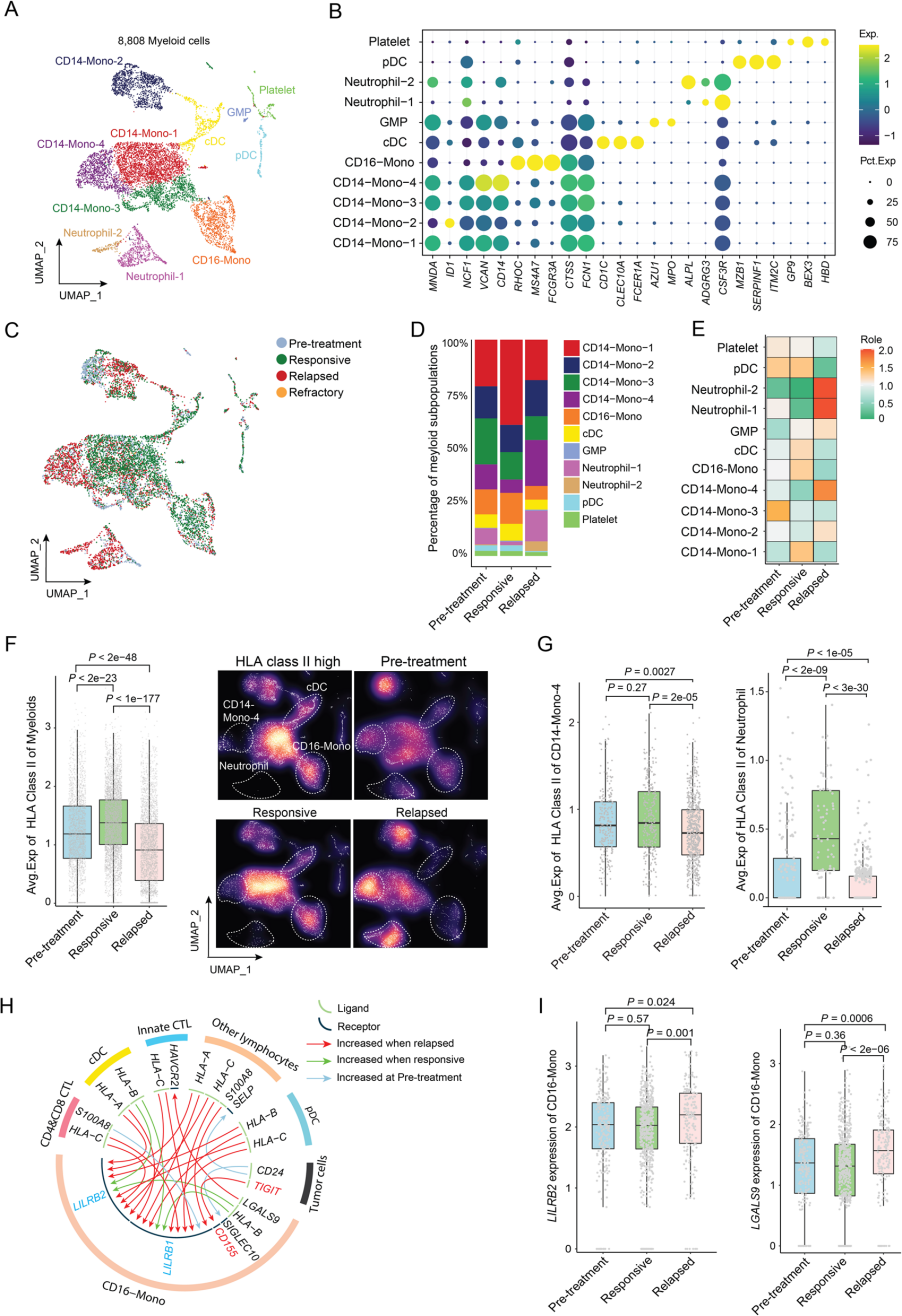
Subsets of monocytes and neutrophils enriched post relapse with low HLA class II expression. (**A**) UMAP plot of myeloid cells. Each dot indicates an individual cell; color denotes myeloid cell subpopulations. (**B**) Bubble heatmap showing HLA class II genes across myeloid clusters. Dot size indicates fraction of expressing cells, colored according to normalized expression levels. (**C**) UMAP plot of myeloid cells. Each dot indicates an individual cell; color denotes clinical response. (**D**) Bar plots showing distribution of each myeloid cell subset at pre-treatment, responsive, and relapsed stages. (**E**) Heatmap showing the enrichment score of each myeloid cell subset at pre-treatment, responsive, and relapsed stages. (**F**) Left panel, box plots showing average expression of HLA class II genes in myeloid cells. *P* values determined by Mann-Whitney test. Right panel, 2D-density plots showing the distribution of myeloid cells in the UMAP plot of (**A**). Brightness of each dot is determined by how many points are around it. (**G**) Box plots showing average expression of HLA class II genes in CD14-Mono-4 cells and Neutrophils. *P* values determined by Mann-Whitney test. (**H**) Circos plot showing ligand-receptor (L-R) interactions between cell types. Only L-R pairs associated with genes showing statistically significant association with clinical response are shown. (**I**) Box plots showing expression of *LGALS9* and *LILRB2* in CD16^+^ Mono cells. *P* values determined by Mann-Whitney test

To dissect relapse-associated myeloid cell enrichment (Fig. 1F, right panel), we first checked the myeloid cellular composition. The myeloid cells can be sub-clustered into 11 subsets including CD14^+^ monocytes (CD14-Mono-1, − 2, − 3, and − 4), CD16^+^ monocytes, neutrophils (Neutrophil-1 and -2), conventional and plasmacytoid dendritic cells (cDCs and pDCs, respectively), and other types including granulocyte-monocyte progenitor (GMP) cells and platelets (Fig. 3A-B). We next examined changes in myeloid cell compositional alterations pre-BA, during remission, and after relapse (Fig. 3C). While the fraction of subcluster CD14-Mono-1 was increased during remission, CD14-Mono-4 and neutrophils (both subclusters Neutrophil-1 and -2) were markedly increased after relapse (Fig. 3D-E).

For the immune recognition and subsequent cytotoxic killing by T cells, the tumor antigens would first be presented by antigen-presenting cells or tumor cells via human leukocyte antigens (HLA) I/II, and loss of HLA I/II expression impaired anti-tumor immune surveillance [18, 19]. Therefore, we examined HLA I/II expression in these myeloid cells. Overall, HLA II was highly differentially expressed across myeloid cell subsets and across BA treatment stages (Fig. 3F). The myeloid cells after relapse showed the lowest HLA II expression (statistically significant, *P* < 2e-23) (Fig. 3G, left panel). The myeloid cells during remission were distributed in a pattern similar to that of HLA II^high^ cells, while the cells after relapse showed an opposite distribution pattern (Fig. 3F, right panel). This difference is also seen within specific subpopulations, for example, CD14-Mono-4, neutrophils, and CD16^+^ monocytes (Fig. 3G and Supplementary Fig. S4A-B). These data suggest that the loss of HLA II expression is common among myeloid cells after BA relapse, and this loss may attenuate antigen presentation to T cells and thus may contribute to less cytotoxic killing.

In contrast, expression of HLA I molecules did not show any correlation with BA relapse. However, when we examined potential cell-to-cell communications using the iTALK algorithm (20), we discovered that the crosstalk between HLA I molecules on various types of immune cells with their phagocytosis checkpoints *LILRB1* and *LILRB2 (*leukocyte immunoglobulin-like receptor B1 and B2) on CD16^+^ monocytes, were greatly increased after relapse (Fig. 3H). Furthermore, the expression of *LILRB2* and *LGALS9* (galectin 9, a ligand of TIM3), was significantly increased in CD16^+^ monocytes after BA relapse (Fig. 3I). Together, these data suggest that not only HLA II-mediated tumor antigen presentation was diminished (due to reduced HLA II expression in myeloid cells), but also the HLA I-mediated tumor antigen presentation was suppressed. Collectively, these data indicate that the overall tumor antigen presentation after BA relapse is greatly reduced and thus potentially attenuated the tumor antigen recognition by cytotoxic T cells.

### Myeloid-derived suppressive cells (MDSCs) are increased after BA relapse

**Fig. 4:**
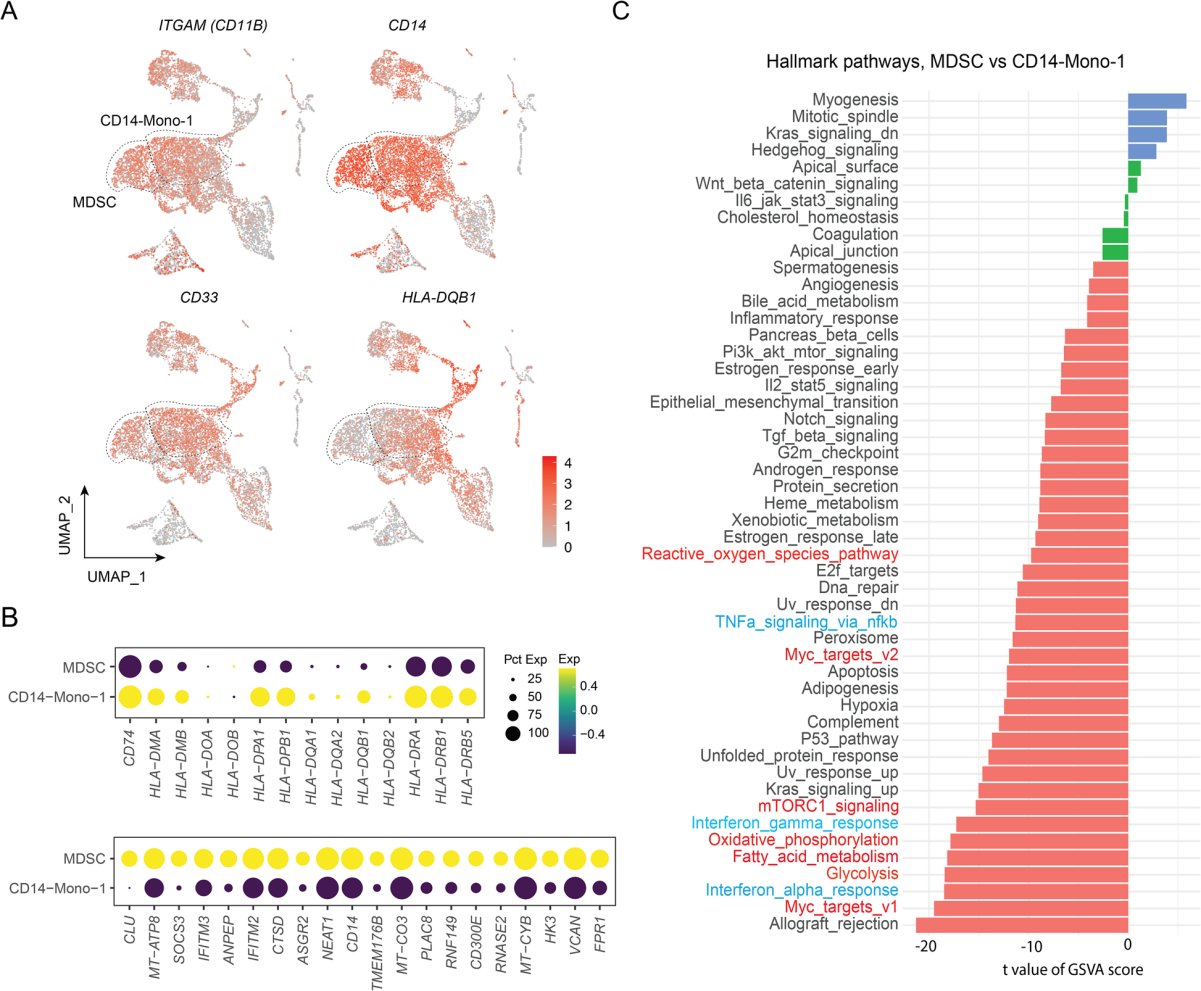
MDSCs post relapse showed remarkable transcriptomic reprogramming. **A** UMAP plots of individual gene expression. Each dot indicates an individual cell; color denotes gene expression intensity. **B** Bubble heatmap showing HLA class II genes across myeloid clusters. Dot size indicates fraction of expressing cells, colored according to normalized expression levels. **C** Gene set enrichment analysis of cancer hallmarks comparing MDSC vs CD14-Mono-1

Interestingly, the myeloid cells in the CD14-Mono-4 subcluster showed similar characteristics to previously-described mononuclear myeloid-derived suppressor cells (M-MDSCs) [20], including low expression of many HLA class II molecules and the expression of known marker genes, such as CD11b^+^, CD14^+^, CD33^+^, and CD15^−^ (Fig. 4A-B). Analysis of differentially expressed genes (DEGs) showed that these MDSCs expressed high levels of the activation markers *CLU* (clusterin), *VCAN* (vercican), *VSIR* (V-set immunoregulatory receptor) and *PIM1* (PIM-1 proto-oncogene, serine/threonine protein kinase), and of the MDSC surface marker *ASGR2* (asialoglycoprotein receptor 2) *(*Fig. 4B). Compared to the remission-associated CD14-Mono-1 subcluster, relapse-associated MDSCs (or CD14-Mono-4) showed transcriptomic reprogramming of hallmark pathways, especially on MYC targets and metabolism-relevant pathways (including glycolysis, fatty acid metabolism, and oxidative phosphorylation) (Fig. 4C and Supplementary Fig. S4C).

### MCL cells acquire TIGIT expression and lose expression of CD19 and HLA-II molecules after BA relapse

**Fig. 5:**
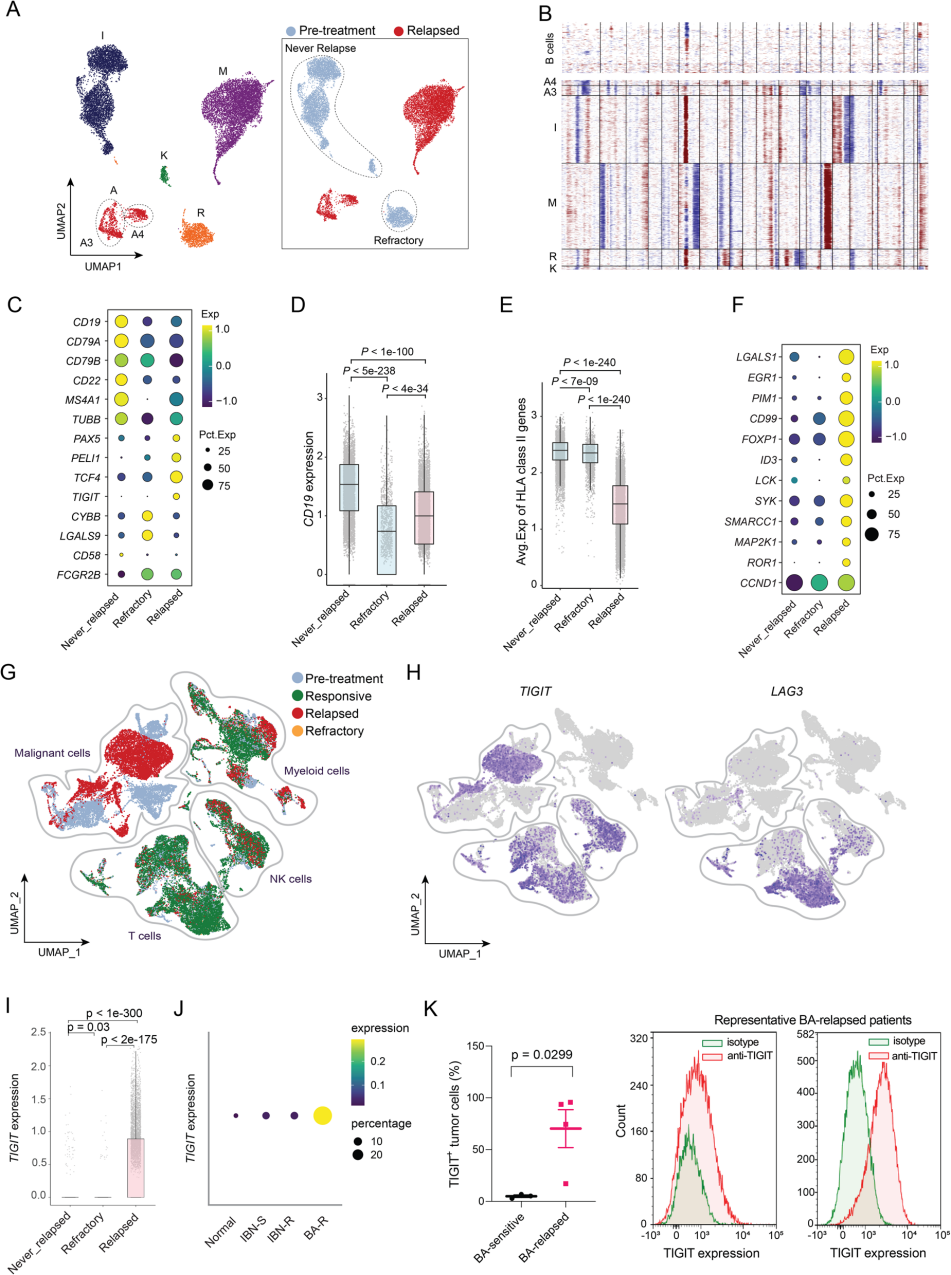
Overexpression of TIGIT and other DEGs in tumor cells associated with BA resistance. (**A**) UMAP plot of tumor cells. Each dot indicates an individual cell; color denotes patients (left) or treatment history (right). (**B**) Inferred copy number based on scRNA-seq data. B-cells from healthy donors are used as normal reference for CNV (Copy Number Variation) inference of malignant cells. (**C**) Bubble heatmap showing top DEGs across distinct groups. Dot size indicates fraction of expressing cells, colored according to normalized expression levels. (**D-E**) Box plots showing average expression of CD19 (**D**) and HLA class II genes (**E**) for single cells. *P* values determined by Mann-Whitney test. (**F**) Bubble heatmap showing expression of top upregulated genes in BA-relapsed tumor cells. (**G**-**H**) UMAP plots of single cells color-coded by the response to BA therapy (**G**) or by expression of individual genes *TIGIT* and *LAG3* (**H**). (**I**) Box plot showing *TIGIT* expression in single B cells. *P* values determined by Mann-Whitney test. (**J**) Bubble heatmap showing expression of *TIGIT* in normal B cells from healthy donors (*n* = 2), and MCL cells from ibrutinib-sensitive (IBN-S, *n* = 4), ibrutinib-resistant (IBN-R, *n* = 17) or BA-resistant (BA-R, *n* = 6) parents. (**K**) TIGIT expression is acquired on the cell surface of MCL tumor cells from BA-relapsed patients (*n* = 4) compared to BA-sensitive patients (*n* = 3) (left panel). Histogram plots (right panels) show cell surface TIGIT expression on MCL tumor cells from two representative BA-relapsed patients

To understand the contribution of tumor intrinsic factors to BA resistance, we applied the inferCNV algorithm to infer large-scale copy number alterations from the scRNA-seq data. The majority of MCL cells were from a PB sample (R1, refractory, post-BA infusion), two apheresis samples (A4 and M4, relapsed, after relapse), and three non-PB samples including excisional lymph node biopsy (A3, relapsed, after relapse), spleen (I2, responsive, pre-treatment), and bone marrow (K0, responsive, pre-treatment) (Fig. 5A). The tumor cells clustered depending on sample source, indicating a degree of inter-tumor heterogeneity (Fig. 5B). Many cell surface molecules were drastically downregulated on MCL cells after relapse (Supplementary Fig. S5A). These include *CD19*, the target of BA (Fig. 5C-D), and other cell surface markers, *CD79A, CD79B, CD22,* and *CD20* (*MS4A1*). Similar to the myeloid cells, HLA II molecules showed markedly lower expression on tumor cells after relapse (Fig. 5E). In contrast, oncogenes including *TCF4* [21], *PIM1* [22], and *ROR1,* and the B-cell inhibitory checkpoint molecule *FcγRIIB* [23], were all elevated in relapsed samples (Fig. 5C and F). Furthermore, the checkpoint molecule *TIGIT* that was found to be acquired in T cells and NK cells (Fig. 2H) was also highly expressed on MCL cells after relapse (Fig. 5G-I), compared to that observed at pretreatment. In contrast, expression of the checkpoint molecule *LAG3* was restricted to T cells and a small fraction of NK cells, with no apparent expression on MCL cells or myeloid cells (Fig. 5H). To validate this, we checked *TIGIT* expression in MCL cells from other patient cohorts. *TIGIT* expression was rarely detected in normal B cells (mean = 0.0144) serving as negative controls, and slightly increased in ibrutinib-sensitive (mean = 0.0287) and ibrutinib-resistant MCL cells (mean = 0.0364). In contrast, *TIGIT* expression was markedly increased in MCL cells after BA relapse (mean = 0.103, *P* = 2.22e-16) (Supplementary Fig. S5B), with larger fractions of MCL cells expressing *TIGIT* (Fig. 5J). When we checked the cell-to-cell communication, we found that expression of *CD155*/*PVR* was increased in CD16^+^ monocytes after relapse and the crosstalk between TIGIT molecules (on MCL cells) with its ligand CD155 (on CD16^+^ monocytes) was greatly increased after relapse (Fig. 3H). This suggested that acquired TIGIT expression on MCL cells could create an opportunity for these cells to directly suppress cytotoxic effector cells via the TIGIT-CD155-CD226-axis. Furthermore, TIGIT was validated to be expressed on MCL tumor cells after BA relapse (Fig. 5K) and TIGIT expression on tumor cells suppressed IFNγ production by T cells (Supplementary Fig. S5C). These data suggested that targeting TIGIT might prevent CAR T relapse and improve patient outcomes. Unlike the case in these BA-relapsed patients, TIGIT did not appear to be expressed in MCL tumor cells in the BA-refractory patient (R); in contrast, elevated expression of other checkpoint molecules, *LAGLS9* and *CYBB,* was detected in MCL tumor cells from patient R (Supplementary Fig. S5D).

### Cytokines, chemokines, and soluble receptors in plasma correlate with BA relapse

**Fig. 6:**
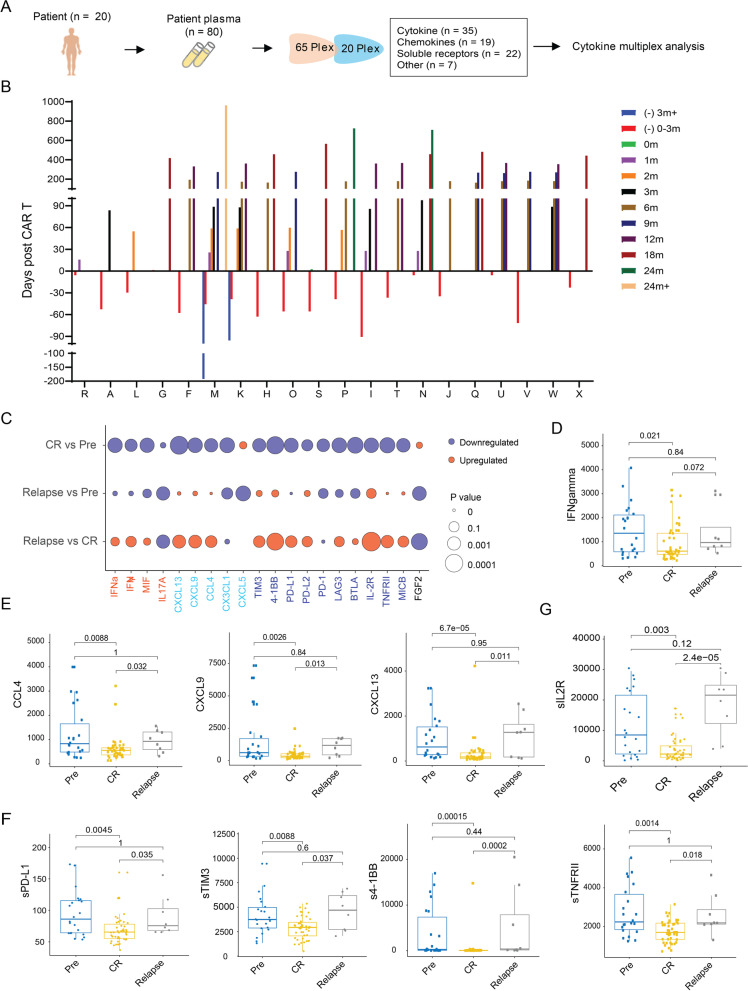
Cytokines, chemokines, and soluble receptors in blood correlating with BA relapse. (**A**) Schematic design for cytokine multiplex. (**B**) Longitudinal collection time points for patient plasma samples from each patient. (**C**) Heatmap of log value of *P* value for cytokines, chemokines, soluble checkpoint receptors and other receptors. (**D-G**) Individual dot plots of serum cytokines (**D**), chemokines (**E**), soluble checkpoint receptors (**F**), and other soluble receptors (**G**) that are statistically significantly altered during BA-remission or after BA-relapse

To evaluate the extracellular milieu potentially associated with BA relapse, we performed high-throughput cytokine profiling on patient plasma samples (*n* = 80) collected longitudinally from 20 patients. These included the 15 patients comprising the scRNA-seq cohort plus five additional patients (patients T-X) who received BA (Fig. 6A-B). In total, we included 83 analytes including cytokines (*n* = 35), chemokines (*n* = 19), soluble receptors (*n* = 22), and others (*n* = 7) (Supplementary Table S3), that are functionally important for cell-to-cell communication during immune and inflammatory responses.

Chemokines CCL4, CXCL9, and CXCL13 were statistically significantly (*P* < 0.01) reduced only during BA remission and apparently returned to baseline levels after relapse (Fig. 6C and E). The CCL4/CCR5 axis, CXCL9/CXCR3-axis, and CXCL13/CXCR5-axis have previously been shown to promote cancer progression and metastasis [24–28]. So, we examined the expression of *CCR5*, *CXCR3*, and *CXCR5* in T cells. *CXCR3* expression was detected in exhausted CD8^+^ cells, proliferating T cells, and CD4+ memory cells, but not in other T-cell subsets (Fig. 2B). Furthermore, relative to BA-remission, the BA-relapse expression of CXCR3 was elevated in overall CD3+ T cells (*P* < 0.0001), particularly in exhausted CD8+ T cells (*P* < 0.05). However, this increase was not seen in proliferating T cells or CD4^+^ memory cells (Supplementary Fig. S6A). In addition, no apparent correlation of IFNγ levels with remission or relapse were observed in MCL (Fig. 6D), which is distinct from CAR T-cell treated patients with DLBCL [5].

A large fraction (10/22) of the soluble forms of the tested cell surface proteins were statistically significantly (*P* < 0.05) reduced in patient plasma during BA remission relative to pre-BA (Fig. 6F-G). These included six soluble checkpoint inhibitors (sPD-L1, sPD-L2, sPD-1, sTIM3, sLAG3, sBTLA, and s4-1BB) and three other cell surface proteins (sIL-2R, sTNFRII, and sMICB) (Fig. 6F-G). Interestingly, five of these, sPD-L1, sTIM-3, s4-1BB, sIL-2R, and sTNFRII, were statistically significantly (*P* < 0.05) upregulated after relapse compared to during BA remission (Fig. 6F-G). It has been reported that serum levels of soluble immune checkpoint-related proteins can serve as predictors of tumor progression and recurrence survival in cancer patients [29]. It has also been shown that soluble TIM3 [30], PD-L1, and PD-L2 levels [31] correlate with poor patient survival. Together, these data suggest that elevated serum levels of these soluble checkpoint molecules could serve as prognostic markers to predict CAR-T relapse.

sIL2R was the most statistically significantly (*P =* 0.0000024) elevated soluble protein in plasma after relapse (Fig. 6G). Ten of thirteen patients (77%) showed statistically significantly (*P* = 0.003) reduced blood sIL2R levels during BA remission compared to pre-BA, and all four relapsed patients had statistically significantly (*P* = 2.4e-5) elevated sIL2R levels, compared to that observed during BA remission (Supplementary Fig. S6B). Increased plasma sIL2R was further confirmed in patient F after relapse by independent ELISA assay (Supplementary Fig. S6C, right panel). In contrast, IL-2 levels were only slightly increased during relapse (Supplementary Fig. S6C, left panel).

To identify the T cell subset(s) that correlated with elevated sIL2R, we expanded T cells from healthy PBMCs and purified CD3^+^ T cells from them, which were further separated into CD4^+^CD25^+^, CD4^+^CD25^−^, and CD8^+^ T cell subsets. P/I induced robust production of both IL-2 and IFNγ in all four types. sIL2R in cell supernatants was detected at much higher levels in CD3^+^ cells and CD8^+^ cells than in CD4^+^CD25^+^ or CD4^+^CD25^−^ cells, and further increased upon P/I stimulation for 24 hours (Supplementary Fig. S6D). High baseline levels of IL2R alpha chain (IL2Rα) or CD25 on the cell surface were detected in all cell types except CD4^+^CD25^−^ cells, which serve as a negative control. Cell surface CD25 expression was reduced upon P/I stimulation in CD3^+^ cells and CD8^+^ cells, but not in CD4^+^CD25^+^ cells (Supplementary Fig. S6D). These data suggested that sIL2R originated mainly from CD8^+^ cells.

To understand the role of sIL2R in regulating T-cell function, we stimulated the T cells collected from patient F at pre-BA and after relapse with IL-2, sIL2R alone, or the two in combination. The cells at pre-BA were able to expand upon stimulation by IL-2 or sIL2R alone, and their combination further enhanced cell growth (Supplementary Fig. S6E-F). However, the cells collected after relapse at both time points (relapse_1 and relapse_2) failed to respond to IL-2 or sIL2R alone, and the combined treatment actually reduced cell growth (Supplementary Fig. S6E-F). These data suggest that elevated sIL2R in relapsed patients may contribute to therapeutic resistance by inhibiting T-cell expansion.

## Discussion

With the rapid development of CAR T-cell therapeutics in hematologic malignancies, emerging resistance and its mechanisms have been increasingly reported in the past few years [5, 7, 8, 11–14]. Those studies focused primarily on the resistance mechanisms involving CAR T-cell products pre-infusion and CAR T cells post-infusion, but very little on the endogenous T cells within the TME. Furthermore, resistance mechanisms have been reported for DLBCL and other hematologic malignancies, but not yet for MCL. It is unknown whether all or any of these resistance mechanisms will apply to MCL. Previous studies showed evidence that MCL has disease-specific mechanisms that confer malignancy and ibrutinib resistance [32, 33]. Therefore, one would expect that this scenario would also apply to CAR T-cell resistance. Indeed, in this study, we revealed several factors that are uniquely associated with CAR T-cell relapse in MCL, or not yet reported for other hematologic malignancies. These include: (1) endogenous T-cell suppression; (2) acquired expression of the checkpoint molecule *TIGIT* in CTLs; (3) acquired TIGIT expression and reduced expression of HLA-II molecules in tumor cells; (4) increased MDSCs and neutrophils; and (5) elevated soluble forms of checkpoint molecules sPD-L1, sTIM3, s4-1BB, and the receptors sIL-2R and sTNFRII, as well as chemokines CCL4, CXCL9, and CXCL13. Among these, TIGIT is the central player in BA cell therapy suppression and disease relapse in MCL.

In this study, we discovered that exhaustion and depletion of endogenous T cells are associated with BA relapse. Distinct from DLBCL [34], *TIM3*, *PD-1*, and *PD-L1* were barely detectable during all treatment stages in MCL. Instead, *TIGIT* expression is the predominant checkpoint molecule that is acquired in CTLs and is associated with relapse in MCL. This is not the case for patients with DLBCL who failed axicabtagene ciloleucel (AC) CAR T-cell therapy [34], even though AC and BA share the same CAR T construct. In absence of TIGIT, CD226 – an activation receptor on T cells or NK cells – binds to its ligand CD155 to activate the cytotoxic function of T cells or NK cells. However, when expressed on these cells, TIGIT binds to the ligand CD155 with much higher affinity than CD226, therefore outcompeting CD226 in binding to CD155 and thus suppressing the cytotoxic functions of T cells or NK cells. In this study, we detected higher fractions of TIGIT-expressing CTLs after relapse in MCL, which may explain why the cytotoxic score of these CTLs after relapse is noticeably lower than those during remission. TIGIT was just reported to be a novel marker expressed on CD8 CAR T cells and associated with CAR T-cell exhaustion in patients with non-Hodgkin’s lymphoma [35]. However, whether TIGIT is expressed on endogenous T cells was not addressed. In addition to endogenous T cells and NK cells, we observed that *TIGIT* is expressed in MCL cells after relapse not only at higher levels, but also with higher fractions, which is absent in those at pre-treatment. This demonstrates that *TIGIT* expression is acquired by MCL cells after BA relapse, and this has not yet been reported in any patients with hematologic malignancies or other cancer types after CAR T-cell therapy.

Tumor-intrinsic expression of TIGIT has been reported in patients with colorectal cancer, and was shown to promote tumor progression by competing with CD226 in binding to CD155 [36]. This may also apply to TIGIT-expressing MCL cells. By acquiring TIGIT expression, MCL cells may evade tumor immune surveillance via the TIGIT-CD155-CD226 axis to suppress the cytotoxic function of T and NK cells. Indeed, based on cell-to-cell communication analysis, the interaction between TIGIT on tumor cells with CD155 expressed on CD16^+^ monocytes was markedly increased after relapse. Therefore, it will be of great interest and importance to investigate the potential for targeting TIGIT both as a tumor-intrinsic factor and as a tumor-extrinsic factor using antibody-based immunotherapy to prevent TIGIT-mediated T-cell suppression and immune escape. This TIGIT targeting approach will have two benefits - one on tumor cells and the other on cytotoxic immune cells - allowing CD226 to bind to CD155 to reactivate T cells and NK cells for anti-tumor cytotoxic killing.

Recruitment and expansion of tumor-associated suppressive myeloid lineages such as MDSCs have been increasingly recognized to confer tumor immune evasion and promote therapeutic resistance to CAR T-cell therapies as well as to previous therapies [37–40]. A recent study revealed a higher percentage of M-MDSCs before, but not after, axicabtagene ciloleucel treatment, associated with no durable response in DLBCL [5]. In contrast, we observed higher percentages of MDSCs associated with BA relapse in MCL (Fig. 3D-E). These MDSCs expressed high levels of MDSC activation markers *CLU*, *VCAN, VSIR,* and *PIM1*. CLU selectively promotes MDSC survival [41], and VCAN promotes tumor cell growth and metastasis when secreted by M-MDSCs [42, 43]. High expression of *VSIR* mediates MDSC suppression of T-cell responses in patients with acute myeloid leukemia [44]. PIM1 regulates lipid oxidative metabolism to support the suppression function of MDSCs [45]. Together, expression of these activation markers suggests that these MDSCs are active for immune suppression after BA relapse in MCL. It has been suggested that TIGIT expressed on NK cells is critical for MDSC-mediated immune suppression in NK cells [46]. A similar mechanism may also apply to TIGIT-expressing NK cells and T cells in MCL. If this is the case, the aforementioned TIGIT targeting approach may have one more benefit by rescuing TIGIT-expressing T cells and NK cells from MDSC-mediated immune suppression. This requires further investigation.

## Conclusions

In this study, we discovered multiple tumor-intrinsic and -extrinsic factors that are associated with T-cell suppression and BA-relapse. The acquired expression of the checkpoint molecule TIGIT in not only cytotoxic lymphocytes but also MCL cells is the central mechanism leading to therapeutic relapse. Together, our data suggest that co-targeting TIGIT may prevent CAR T relapse and thus promote long-term progression-free survival.

## Supplementary Information

**Additional file 1: Supplementary Fig. S1.** Cellular composition of immune cells in tumor microenvironment. **Supplementary Fig. S2.** Endogenous T cell clones expanded during responsive stage but depleted post relapse. **Supplementary Fig. S3.** Endogenous T cells post relapse are functionally deficient. **Supplementary Fig. S4.** Checkpoint inhibitors, HLA II molecules and hallmark pathways in monocyte subsets. **Supplementary Fig. S5.** Expression of cell surface genes and enriched hallmark pathways in MCL tumor cells. **Supplementary Fig. S6.** CXCR3 is overexpression in exhausted CD8 T cells and sIL2R and IL-2 failed to induce ex vivo cell expansion of PBMC collected post relapse. **Supplementary Table S1.** Summary of clinical characteristics of 15 patients with MCL. **Supplementary Table S2.** Summary of clinical characteristics for patients with MCL. **Supplementary Table S3.** Analytes included in the 65-plex and 20-plex assays for cytokine profiling.

## Data Availability

The datasets used and/or analyzed during the current study are available from the corresponding author on reasonable request.

## Abbreviations

AC: Axicabtagene ciloleucel
ASGR2: Asialoglycoprotein receptor 2
ASH: American Society of Hematology
BA: Brexucabtagene autoleucel
CAR: Chimeric antigen receptor
CCL4: C-C motif chemokine ligand 4
CCR5: C-C motif chemokine receptor 5
CD40L: CD40 ligand
cDCs: Conventional dendritic cells
CLU: Clusterin
CTLA4: Cytotoxic T-lymphocyte associated protein 4
CTLs: Cytotoxic T cells
CXCL9: C-X-C motif chemokine ligand 9
CXCL13: C-X-C motif chemokine ligand 13
CXCR3: C-X-C motif chemokine receptor 3
CXCR5: C-X-C motif chemokine receptor 5
DLBCL: Diffuse large B-cell lymphoma
ELISA: Enzyme-linked immunosorbent assay
GMP: Granulocyte-monocyte progenitor cells
GNLY: Granulysin
HLA: Human leukocyte antigen
IFNγ: Interferon gamma
KLRD1: Killer cell lectin-like receptor D1
LAG3: Lymphocyte-activation protein 3
LILRB1: Leukocyte immunoglobulin like receptor B1
LILRB2: Leukocyte immunoglobulin like receptor B2
LGALS9: Galectin 9
MCL: Mantle cell lymphoma
MDSC: Myeloid-derived suppressor cells
M-MDSC: Monocytic myeloid-derived suppressor cells
MS4A1: Membrane-spanning 4-domains A1
NK: Natural killer
P/I: Phorbol 12-myristate 13-acetate/ionomycin
PBMCs: Peripheral blood mononuclear cells
PDCD1: Programmed cell death 1
pDCs: Plasmacytoid dendritic cells
PIM1: PIM-1 proto-oncogene, serine/threonine protein kinase
PVR: PVR cell adhesion molecule
ROR1: Receptor tyrosine kinase-like orphan receptor 1
sBTLA: Soluble B and T lymphocyte associated
scTCR-seq: Single-cell T-cell receptor (TCR) repertoire analysis
sIL-2R: Soluble IL-2 receptor
sPD-L1: Programmed death-ligand 1
ssGSEA: Single-sample gene set enrichment analysis
sTIM3: Soluble T cell immunoglobulin mucin 3
sTNFRII: Soluble tumor necrosis factor receptor type II
TCF4: Transcription factor 4
TCR: T cell receptor
TIGIT: T cell immunoreceptor with Ig and ITIM domains
TME: Tumor microenvironment
Tregs: Regulatory T cells
VCAN: Versican
VSIR: V-set immunoregulatory receptor
scRNA-seq: Single-cell RNA sequencing
