# Supplementary material for: TIGIT is the central player in T-cell suppression associated with CAR T-cell relapse in mantle cell lymphoma
Source: Mol Cancer. 2022 Sep 26;21:185. doi: 10.1186/s12943-022-01655-0 (PMC9513944; doi:10.1186/s12943-022-01655-0)
Supplement: Supplementary file 1 — Additional file 1: Supplementary Fig. S1. Cellular composition of immune cells in tumor microenvironment. Supplementary Fig. S2. Endogenous T cell clones expanded during responsive stage but depleted post relapse. Supplementary Fig. S3. Endogenous T cells post relapse are functionally deficient. Supplementary Fig. S4. Checkpoint inhibitors, HLA II molecules and hallmark pathways in monocyte subsets. Supplementary Fig. S5. Expression of cell surface genes and enriched hallmark pathways in MCL tumor cells. Supplementary Fig. S6. CXCR3 is overexpression in exhausted CD8 T cells and sIL2R and IL-2 failed to induce ex vivo cell expansion of PBMC collected post relapse. Supplementary Table S1. Summary of clinical characteristics of 15 patients with MCL. Supplementary Table S2. Summary of clinical characteristics for patients with MCL. Supplementary Table S3. Analytes included in the 65-plex and 20-plex assays for cytokine profiling. [file 12943_2022_1655_MOESM1_ESM.docx]

**Supplementary Data**

**Supplementary Methods**

**TCR V(D)J sequence assembly, paired clonotype calling, and integration with scRNA-seq data.** Cell Ranger v3.0.2 for V(D)J sequence assembly was applied for TCR reconstruction and paired TCR clonotype calling. The CDR3 motif was located, and the productivity was determined for each single cell. The clonotype landscape was then assessed and the clonal fraction of each identified clonotype was calculated. The TCR clonotype data was then integrated with the T-cell phenotype data inferred from single-cell gene expression analysis based on the shared cell barcodes.

***T cell proliferation and expansion from PBMCs from patients and healthy donors.***

PBMCs from patients and healthy donors were purified by Ficoll-Paque Plus density gradient centrifugation before storage in liquid nitrogen. The cryopreserved PBMCs were thawed and cultured in RPMI-1640 medium containing 1% penicillin/streptomycin, 25 mM 4-(2-hydroxyethyl)-1piperazineethanesulfonic acid (HEPES), and 10% fetal bovine serum (FBS; Sigma-Aldrich, St Louis, MO) in presence of anti-CD3/CD28-conjugated beads (11131D, Gibco) and IL-2 (200-02 PeproTech) at 20 ng/ml, or in presence of IL-2 and sIL2Rα (223-2A-025/CF, R&D), alone or in combination. The cells were cultured for 5-10 days for expansion and the resulting cells were validated by flow cytometry analysis. For cell proliferation assay, the cells were counted by cell counter (Bio-Rad) every day for 5 days.

***T cell subset separation and enrichment***

The expanded T cells from PBMCs were further separated and enriched into CD3^+^ T cells (130-050-101, Miltenyi Biotec), CD8^+^ cells (130-045-201, Miltenyi Biotec), CD4^+^ cells, CD4^+^CD25^+^ and CD4^+^CD25^−^ cells (130-091-301, Miltenyi Biotec), according to manufacturer’s instructions.

***T cell activation by PMA/Ionomycin***

The cells were cultured in RPMI-1640 medium containing 1% penicillin/streptomycin, 25 mM HEPES, and 10% FBS, and stimulated with PMA (tlrl-pma, InvivoGen) at 50 ng/ml) and ionomycin (inh-ion, InvivoGen) at 100 ng/ml for 6 or 24 hours

***CSFE proliferation Assay***

The cells were first stained with CSFE (C34554, Invitrogen) according to manufacturer’s instructions and cultured in RPMI-1640 medium containing 1% penicillin/streptomycin, 25 mM HEPES, and 10% FBS in presence of indicated stimulators. For cell proliferation assay, the cells were analyzed by flow cytometry to measure the CSFE staining on cell surface.

***Cell imaging***

The cultured cells in 96 wells or 6 wells were imaged with a brightfield microscope.

**Supplementary Figures**

**
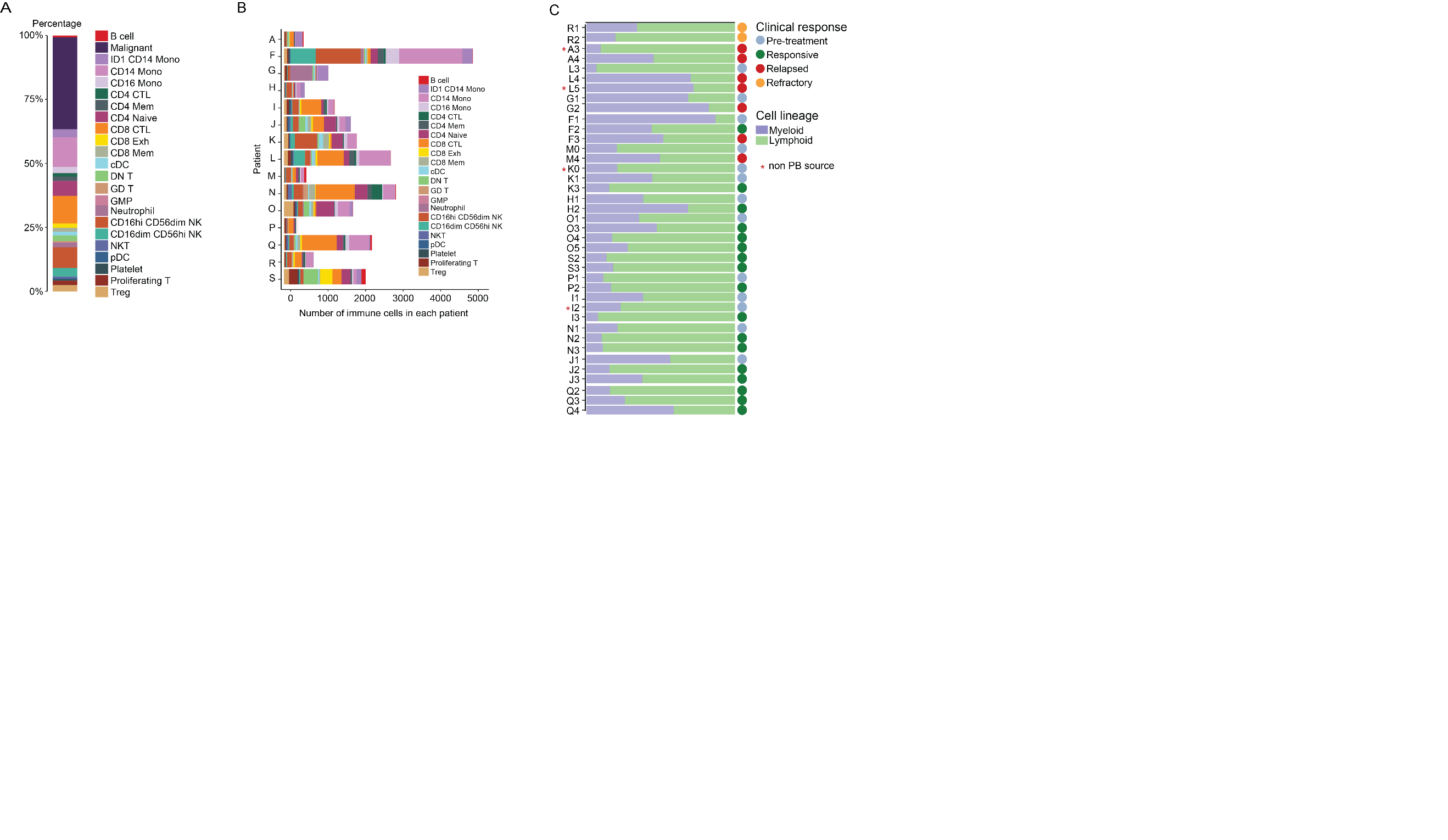
**

**Supplementary Figure S1. Cellular composition of immune cells in tumor microenvironment.**

(**A**) Bar graph showing the distribution of cell types of all single cells that passed quality control test. (**B**) Bar graphs showing the number of immune cells passing QC per patient. (**C**) Bar graph showing the distribution of non-malignant cells from each sample. Relative fractions of myeloid and lymphoid cells are shown. Colored dots are coded by the timepoints of sample collection. Samples not from peripheral blood (PB) are asterisked in red.

**
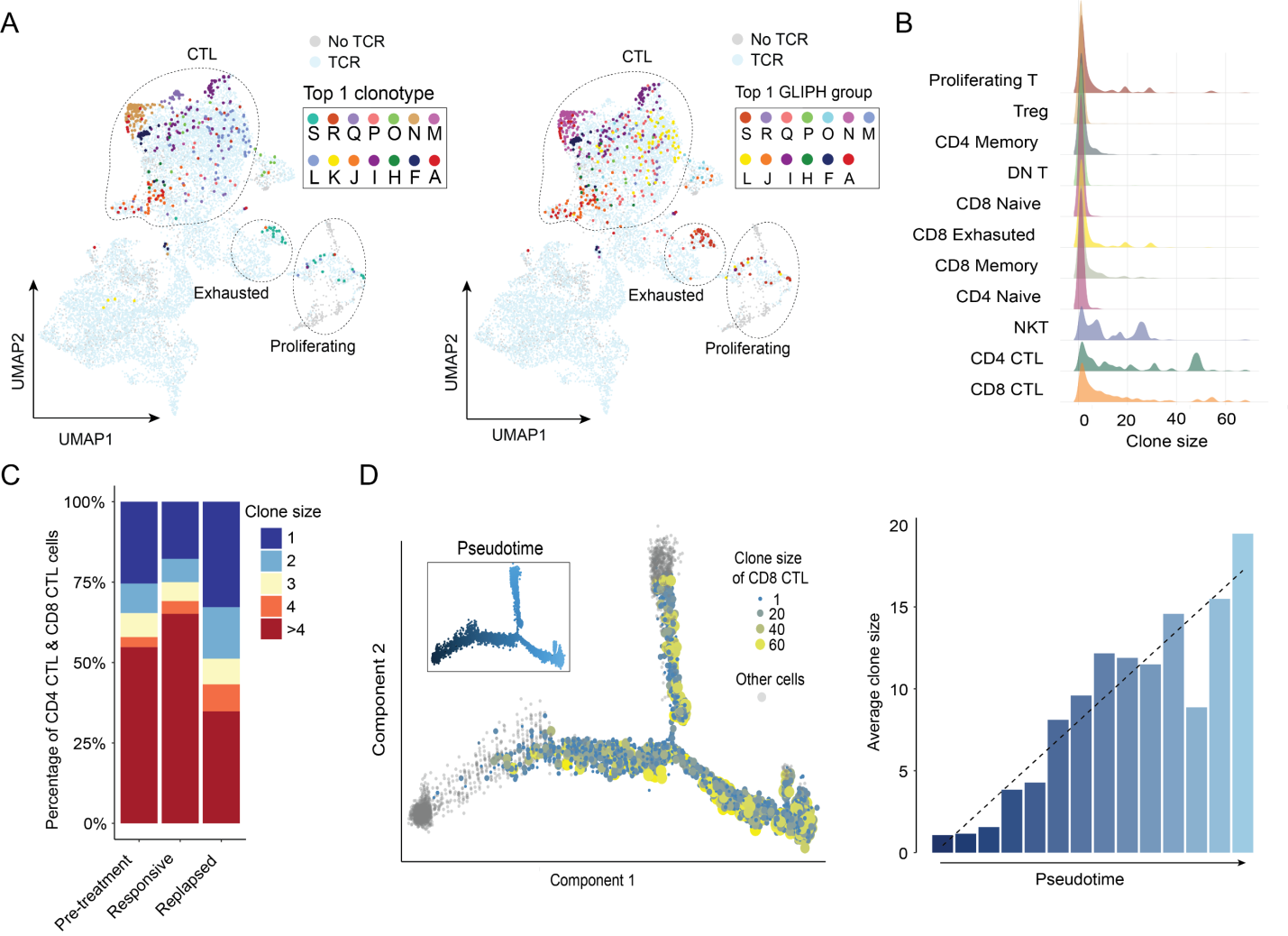
**

**Supplementary Figure S2**. **Endogenous T cell clones expanded during responsive stage but depleted post relapse.**

(**A**) UMAP of T cells colored by selected TCR clonotypes (left) or colored by TCR β chain (TRB) clones belonging to the same TCR specificity (GLIPH) group (right). Only the most abundant clone of each patient is shown. (**B**) Density distribution of clone size of single cells within each cell subset. (**C**) Clone size distribution of combined CTLs (CD4^+^ and CD8^+^) for samples of different treatment stages. Color indicates the size of the clone that single cells belong to. (**D**) Trajectory analysis of CD8^+^ T cells. Left, each dot represents a single cell, with size and color indicating clone size. Right, the variation of average clone size along with pseudotime that is equally divided into bins.

**
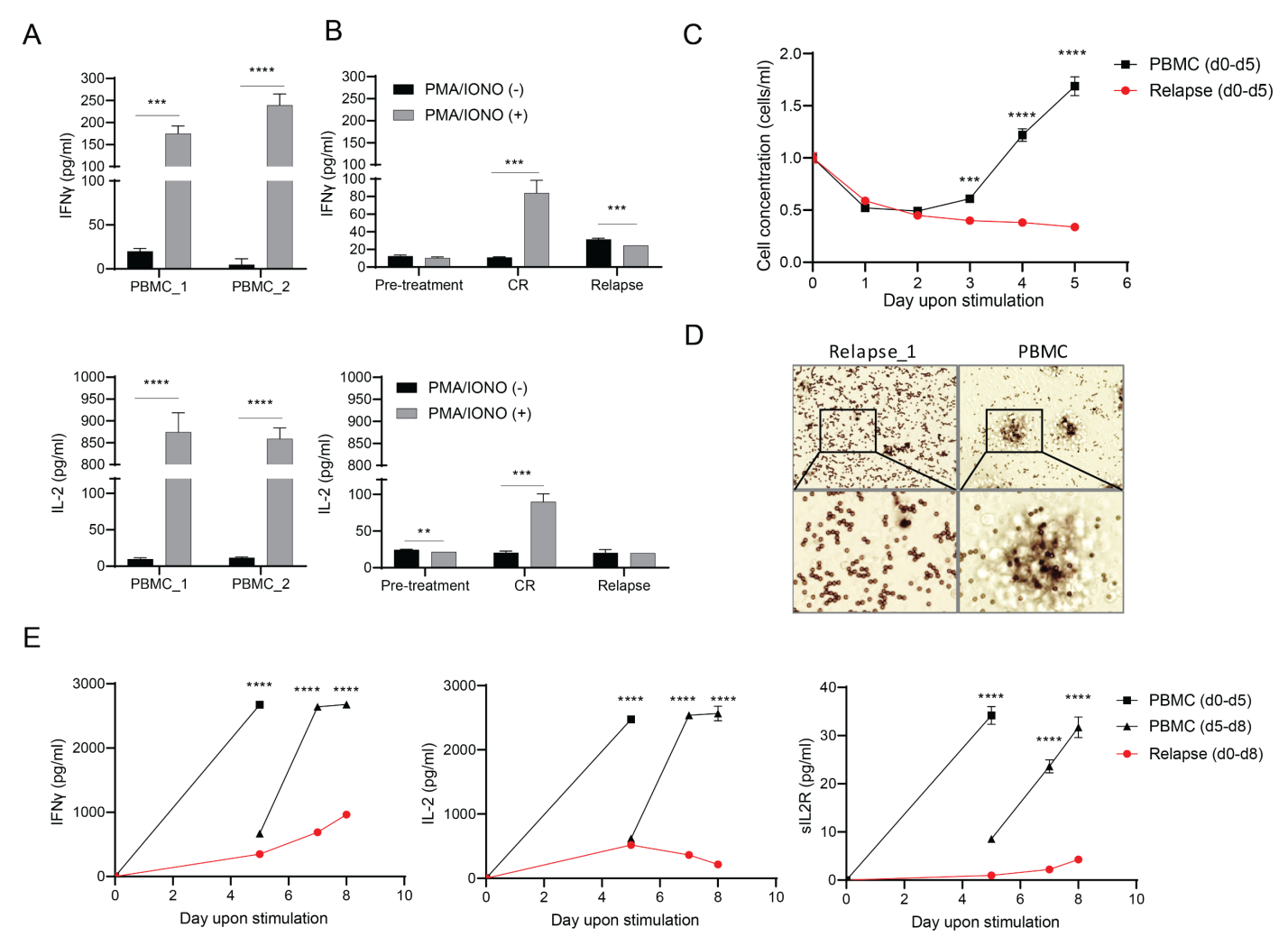
**

**Supplementary Figure S3. Endogenous T cells post relapse are functionally deficient.**

(**A-B**) PMBCs from two healthy donors (**A**) or from patient F at pretreatment, CR, and relapse (**B**) were stimulated with PMA and ionomycin at 50 ng/ml and100 ng/ml, respectively for 24 hours. IL-2 and IFNγ in the culture supernatant were detected by ELISA and plotted. (**C-D**) PMBCs from a healthy donor or from patient F post relapse were stimulated with anti-CD3/CD28 conjugated beads in presence of IL-2 at 20 ng/ml for 5 days. The cell concentrations were determined and plotted in C, with representative images of the cell culture shown in (**D**). (**E**) PMBCs from a healthy donor or from patient F post relapse were stimulated with anti-CD3/CD28-conjugated beads in presence of IL-2 at 20 ng/ml for 5 days. At day 5 the cells expanded from the healthy donor reached confluence and were split 1:5 in culture medium containing IL-2 at 20 ng/ml. The cells were monitored for an additional 5 days. The culture supernatant at day 5 before and after split and at days 7 and 8 were collected and underwent ELISA to detect IFNγ, IL-2, and sIL2R.

**
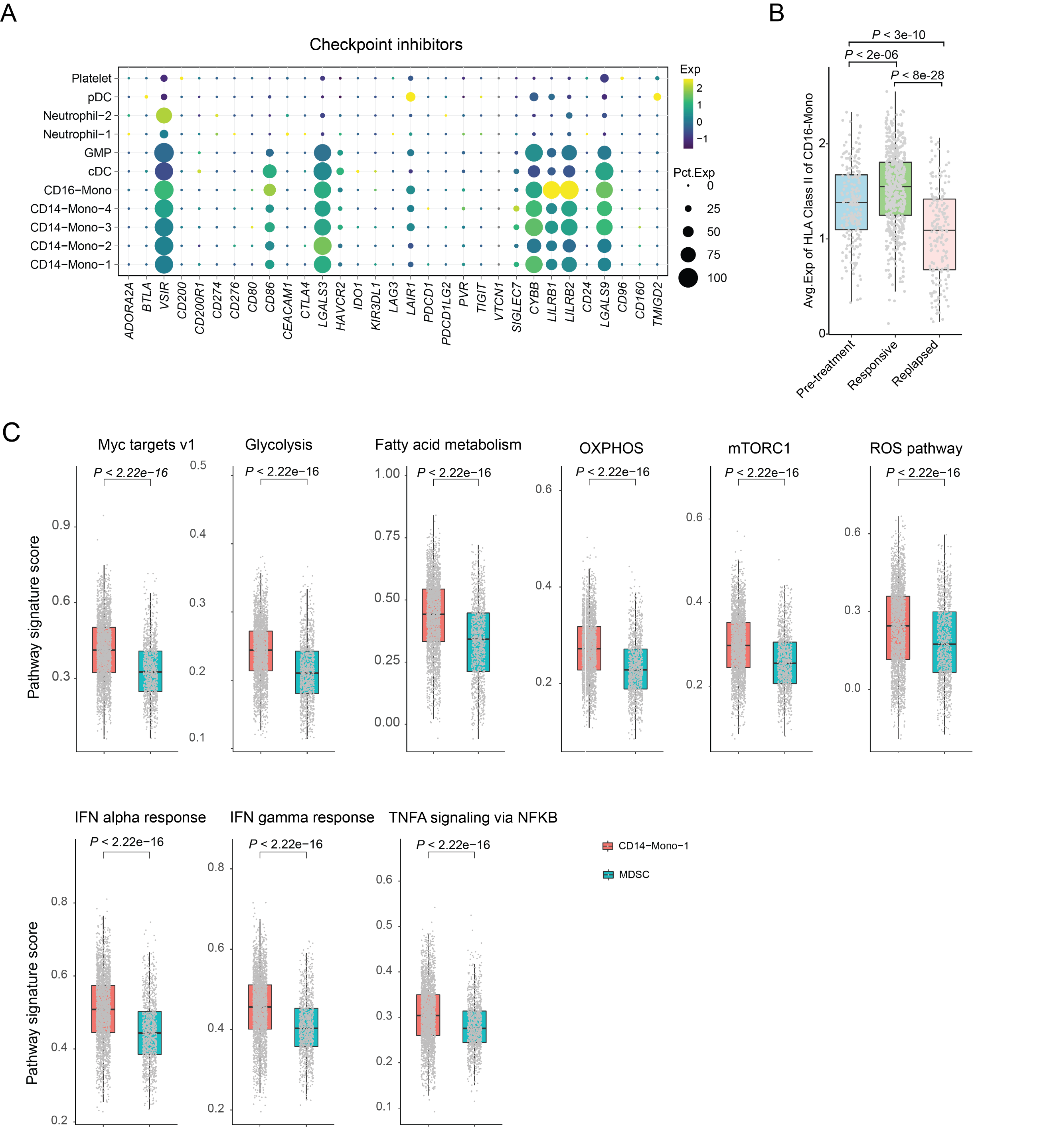
**

**Supplementary Figure S4. Checkpoint inhibitors, HLA II molecules and hallmark pathways in monocyte subsets.**

(**A**) Bubble heatmap showing the top DEGs across myeloid subsets. Dot size indicates the fraction of expressing cells, colored according to normalized expression levels. (**B**) Box plots comparing HLA class II expression in CD16-mono cells at pre-treatment, responsive, and relapsed stages. (**C**) Box plots comparing individual hallmark pathways comparing CD14-mono-1 and MDSC subcultures.

**
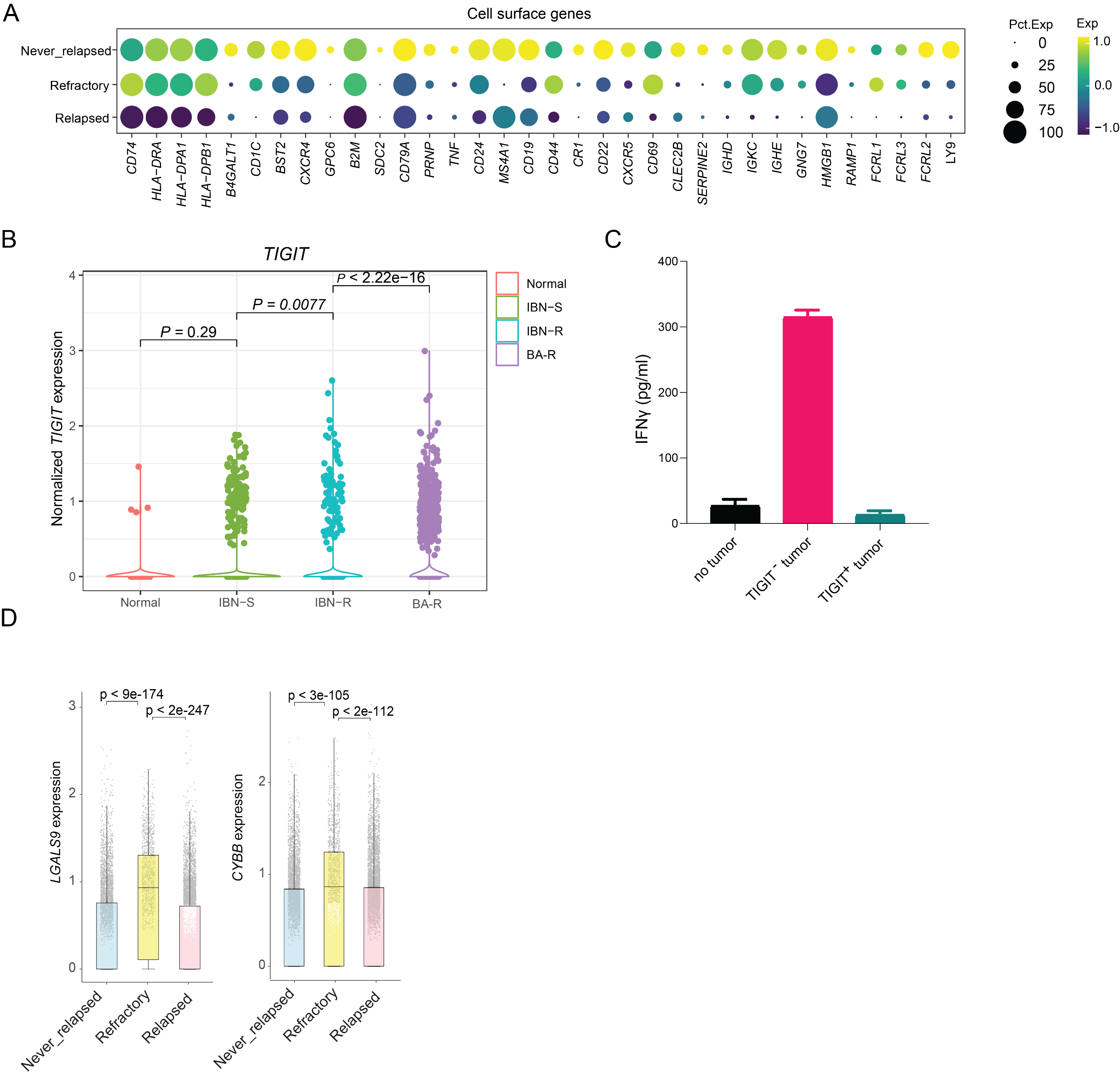
Supplementary Figure S5. Expression of cell surface genes and enriched hallmark pathways in MCL tumor cells.**

(**A**) Bubble heatmap showing expression of cell surface genes in tumor cells across different groups. Bubble size indicates fraction of expressing cells, colored according to normalized expression levels. (**B**) Violin plots showing expression of *TIGIT* in normal B cells from healthy donors (n = 2), and MCL cells from ibrutinib-sensitive (IBN-S, n = 4), ibrutinib-resistant (IBN-R, n = 17) and BA-resistant (BA-R, n = 6) patients. (**C**) TIGIT expression on tumor cells suppressed IFNγ production by T cells. (**D**) Box plots showing average expression of *LGALS9* and *CYBB* for single B cells. *P* values determined by Mann-Whitney test.

**
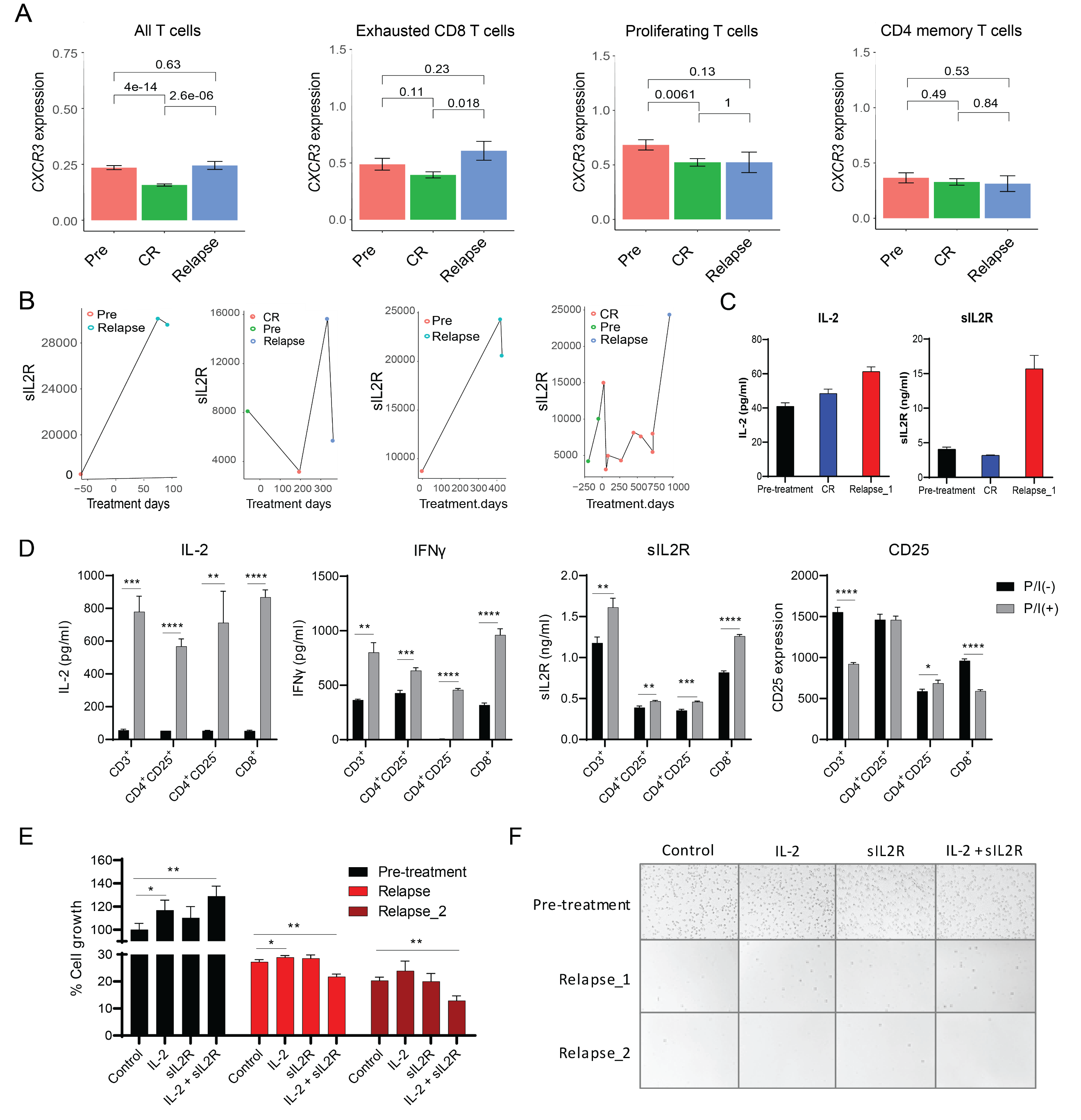
**

**Supplementary Figure S6. CXCR3 is overexpression in exhausted CD8 T cells and sIL2R and IL-2 failed to induce *ex vivo* cell expansion of PBMC collected post relapse.**

(**A**) Bar graph of CXCR3 expression in T cell subsets. *P* values determined by Mann-Whitney test. (**B**) Dot plots of serum sIL2R at pretreatment, responsiveness or relapse for individual relapsed patients at pretreatment, responsiveness, and relapse. (**C**) IL-2 and sIL-2 detection by independent ELISA assay for samples collected from patient F. (**D**) T cells expanded from a healthy donor for 7 days were separated into CD3^+^, CD8^+^, CD4^+^CD25^+^ and CD4^+^CD25^−^ cell subsets. These cells were then stimulated with PMA/ionomycin for 24 hours. The cell supernatant was collected and subjected to ELISA to detect IFNγ, IL-2, and sIL2R and the cells were harvested and subjected to flow analysis to detect CD25 expression on cell surface. (**E-F**) PMBCs from Patient F at pretreatment and relapse were stimulated with IL-2 (10 ng/ml) and sIL-2R (50 ng/ml), alone or in combination, for 5 days. The cell concentrations were monitored by counting and normalized to the starting concentration and plotted (**E**). Representative images of the cell culture were shown (**F**).

**Supplementary Tables**

**Supplementary Table S1**. Summary of clinical characteristics of 15 patients with MCL.

|  |  |
| --- | --- |
| Median age (range) — yr (range) | 67 (52–74) |
| Male — no. (%) | 15 (100%) |
| Prior line of therapy - median (range) | 3 (1-4) |
| Prior lines of therapy (≥3) — no. (%) | 9 (60%) |
| BTK inhibitor therapy — no. (%) | 15 (100%) |
| Cyclin D1 translocation | 4 (27%) |
| MYC translocation | 1 (7%) |
| Blastoid or pleomorphic morphologic characteristics of MCL | 2 (13%) |
| Extranodal lesions | 5 (33%) |
| CD19 status | 13 (87%) |
| Previous BTK inhibitor therapy — no. (%) | 15 (100%) |
| Ibrutinib | 14 (93%) |
| Acalabrutinib | 3 (20%) |
| Both | 2 (13%) |

**Supplementary Table S2**. Summary of clinical characteristics for patients with MCL.

| **Pt** | **Age** | **Sex** | **# prior therapy** | **Prior BTKi** | **CD19 status** | **P53 mutation/deletion** | **CR days post BA** | **Relapse days post CR** | **Initial response** | **Relapse** | **Diagnosis /Sites** |
| --- | --- | --- | --- | --- | --- | --- | --- | --- | --- | --- | --- |
| **R** | **53** | **M** | **3** | **yes** | **Positive** | **ND** | **24** | **49** | **Refractory responder** | **PD** | **Increasing SUV tonsils, cervical, and hilar mass** |
| **A** | **55** | **M** | **1** | **yes** | **Positive** | **Negative** | **26** | **333** | **Fast responder** | **Fast relapse** | **Splenomegaly, Multiple LN** |
| **L** | **62** | **M** | **3** | **yes** | **Positive** | **Negative** | **29** | **35** | **Fast responder** | **Fast relapse** | **Multiple LN** |
| **F** | **69** | **M** | **4** | **yes** | **Positive** | **ND** | **27** | **282** | **Fast responder** | **Slow relapse** | **Multiple LN** |
| **G** | **67** | **M** | **2** | **yes** | **Negative** | **Negative** | **30** | **59** | **Fast responder** | **Fast relapse** | **Multiple LN** |
| **M** | **70** | **M** | **2** | **yes** | **Positive** | **Negative** | **27** | **900** | **Fast responder** | **Slow relapse** | **Multiple LN** |
| **H** | **74** | **M** | **2** | **yes** | **Positive** | **ND** | **29** | **-** | **Fast responder** | **No relapse** | **Multiple LN** |
| **K** | **70** | **M** | **4** | **yes** | **Positive** | **Negative** | **25** | **-** | **Fast responder** | **No relapse** | **Multiple LN** |
| **O** | **58** | **M** | **4** | **yes** | **Positive** | **Negative** | **29** | **-** | **Fast responder** | **No relapse** | **Thrombocytopenia and splenomegaly, LN** |
| **S** | **66** | **M** | **3** | **yes** | **Positive** | **Negative** | **29** | **-** | **Fast responder** | **No relapse** | **Blastoid multiple LN** |
| **I** | **72** | **M** | **2** | **yes** | **Positive** | **ND** | **67** | **-** | **Moderate responder** | **No relapse** | **Multiple LN** |
| **N** | **74** | **M** | **3** | **yes** | **Positive** | **Negative** | **68** | **-** | **Moderate responder** | **No relapse** | **Multiple LN** |
| **P** | **58** | **M** | **3** | **yes** | **ND** | **ND** | **60** | **-** | **Moderate responder** | **No relapse** | **Splenomegaly, Multiple LN** |
| **J** | **52** | **M** | **1** | **yes** | **Positive** | **ND** | **182** | **-** | **Slow responder** | **No relapse** | **Multiple LN** |
| **Q** | **65** | **M** | **4** | **yes** | **Positive** | **Gain** | **272** | **-** | **Slow responder** | **No relapse** | **Blastoid, subcutaneous** |

**Supplementary Table S3.** Analytes included in the 65-plex and 20-plex assays for cytokine profiling.

|  | **65-plex** | **20-plex** |
| --- | --- | --- |
| **Cytokines** | G-CSF (CSF-3), GM-CSF, IFN alpha, IFN gamma, IL-1 alpha, IL-1 beta, IL-2, IL-3, IL-4, IL-5, IL-6, IL-7, IL-8 (CXCL8), IL-9, IL-10, IL-12p70, IL-13, IL-15, IL-16, IL-17A (CTLA-8), IL-18, IL-20, IL-21, IL-22, IL-23, IL-27, IL-31, LIF, M-CSF, MIF, TNF alpha, TNF beta, TSLP | IL-17F, Ferritin |
| **Chemokines** | BLC (CXCL13), ENA-78 (CXCL5), Eotaxin (CCL11), Eotaxin-2 (CCL24), Eotaxin-3 (CCL26), Fractalkine (CX3CL1), Gro-alpha (CXCL1), IP-10 (CXCL10), I-TAC (CXCL11), MCP-1 (CCL2), MCP-2 (CCL8), MCP-3 (CCL7), MDC (CCL22), MIG (CXCL9), MIP-1 alpha (CCL3), MIP-1 beta (CCL4), MIP-3 alpha (CCL20), SDF-1 alpha (CXCL12) | RANTES |
| **Soluble receptors** | APRIL, BAFF, CD30, CD40L (CD154), IL-2R (CD25), TNF-RII, TRAIL (CD253), TWEAK | TIM-3, IL-2R, CD152/CTLA4, LAG-3, BTLA, GITR, PD-1, PD-L1, PD-L2, CD40L (CD154), MICA, MICB, CD137/4-1BB, ULBP-1, ULBP-3, ULBP-4 |
| **Other** | FGF-2, HGF, MMP-1, NGF beta, SCF, VEGF-A | Arginase |
